# Elsberg syndrome in HSV-2 infection

**DOI:** 10.1016/j.idcr.2023.e01714

**Published:** 2023-02-16

**Authors:** Omar Belfaqeeh, Alexandria Markley, Mudita Patel, Brian Markoff, Georgina Osorio

**Affiliations:** Mount Sinai Morningside-West Hospital Center, Icahn School of Medicine at Mount Sinai, New York, NY, United States

**Keywords:** Elsberg syndrome

## Abstract

Elsberg syndrome (ES) is a neuroinflammatory disease that causes acute or subacute lumbosacral radiculitis, with or without myelitis which accounts for approximately 5–10% of cauda equina syndrome and myelitis. We herein present a case of a middle-aged female who recently returned from the Dominican Republic and presented to the emergency room with complaints of a 10-day history of progressive lower extremity sensory changes and weakness preceded by transient bilateral arm pain and neck and head pressure. Based on clinical, radiographic, and serological testing the patient was diagnosed with HSV2 lumbosacral radiculitis (ES). After 21 days of Acyclovir, 5 days of high dose IV methylprednisolone, and one month of inpatient rehab, our patient was discharged home walking with a cane. As ES is poorly defined and rarely reported, it can be unrecognized in patients with acute cauda equina syndrome (CES). Appropriate testing for viral infection in a timely manner facilitates reaching a definitive diagnosis and prompt initiation of treatment, which is essential for resolution of symptoms.

## Background

Elsberg syndrome (ES) is a neuroinflammatory disease that causes acute or subacute lumbosacral radiculitis, with or without myelitis [1], [8]. ES typically presents as cauda equina syndrome (CES), with symptoms of sensory impairment, lower extremity weakness, saddle anesthesia, and urinary and/or bowel incontinence [7], [9]. It is usually associated with infectious causes such as SARS-CoV-2 [1], West Nile Virus [5], Varicella Zoster Virus (VZV) [2], [6], and Herpes Simplex Virus Type 2 (HSV-2), with HSV-2 being the predominate causative pathogen [7]. HSV-2 is dormant in 40% of sacral dorsal root ganglia; when reactivated, virus can spread axonally into the spinal cord [4]. Primary genital infection similarly causes neurologic dysfunction, most commonly in younger patients [9]. Immunocompromised patients such a those with malignancy, HIV, or history of organ transplants are at a greater risk for Herpes Zoster infections, which is a cause of infectious lumbosacral radiculitis and myelitis [3]. Additionally, ES related to VZV should be considered in the differential diagnosis of patients with prolonged poorly controlled T2DM [6]. Treatment with acyclovir, even in cases without a definitive viral cause, is considered beneficial in many cases [7]. Duration of treatment varies and is typically between 10 and 21 days. The use of Steroids in the treatment of ES is debated. Oral steroid tapers or short course high dose IV steroids can be used to help shorten the duration of symptoms [6], [7]. Elsberg syndrome accounts for approximately 5–10% of cauda equina syndrome and myelitis [7] and up to 30% of patients have a recurrence of symptoms within the first year [4].

Below we present a unique case of HSV2 lumbosacral radiculitis (Elsberg Syndrome) diagnosed at a large New York City Hospital Center.

## Case report

Our patient was a 51-year-old female with a past medical history significant for fibromyalgia, right-sided sciatica, right lower extremity deep venous thrombosis, asthma, nephrolithiasis, vertigo, and oral and genital herpes infection who recently returned from Dominican Republic. She presented to the emergency room with complaints of a 10-day history of progressive lower extremity sensory changes and weakness preceded by transient bilateral arm pain and neck and head pressure. Furthermore, she reported being unable to completely void for one day in duration. She described the weakness to be worse in the right leg compared to the left leg, describing it as “leg heaviness” which resulted in a fall at home a day prior to presentation. While she was sitting in the bathroom, she tried to stand but her knees 'buckled' and she fell backward, hitting her head on the floor (denied any loss of consciousness). Her family carried her to the Emergency Department as she was unable to bear weight.

Vital signs on presentation were within normal limits. Physical exam was most notable for reduced power of knee flexors and knee extensors (3/5), dysesthesia to touch and pinprick from mid-thigh distally in stocking distribution bilaterally, absent knee reflexes bilaterally, positive Babinski bilaterally, and no clonus. Given her weakness and urinary hesitancy, a magnetic resonance imaging (MRI) of the cervical, thoracic, and lumbar spine was done to assess for spinal cord pathology and a bladder scan was done to rule out urinary retention. Neurology evaluated the patient for acute progressive lower extremity neuropathic pain and weakness. A lumbar puncture was performed to rule out atypical Guillain Barre syndrome or multiple sclerosis.

**Fig. 1:**
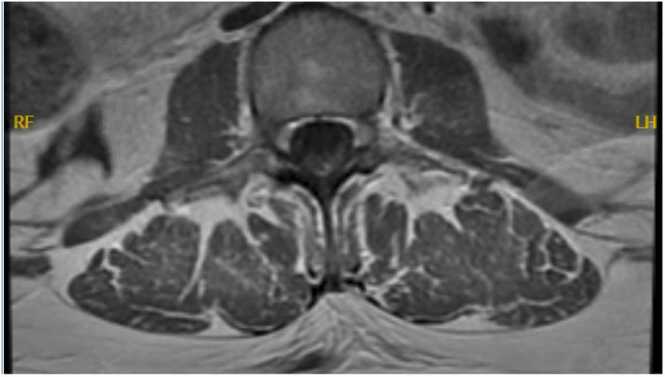
Repeat MRI whole spine w/wo contrast demonstrating a linear enhancement in the dorsal aspect of the thecal sac from L2 to L4.

Complete blood count showed a white blood cell count of 5.1 × 103 cells/mL, hemoglobin of 11.9 g/dL, platelet count of 371 × 103 cells/mL, and eosinophils elevated to 5.7%. The basic metabolic panel was within normal limits. Vitamin B12 level came back low at 290 pg/mL, homocysteine of 11.2, ESR 30.9 and CRP 5.9. Other labs including creatine phosphokinase, aldolase, methylmalonic acid, Lyme titers, copper level, vitamin B12, SSA/SSB (Sjogren Antibodies), were unremarkable. The CSF PCR cytology showed pleocytosis with a WBC count of 360 (100% lymphocytes), although no albumin cytologic dissociation was seen, and protein of 182 mg/dL. CSF PCR was positive for HSV-2. Infectious disease (ID) was consulted and reviewed the MRI spine with radiology, which demonstrated a very faint enhancement of the L4–5 nerve roots. An MRI brain came back within normal limits. An initial electromyography (EMG) study done during the first week of her admission was unremarkable. A repeat MRI of the whole spine 7 days later showed linear enhancement in the dorsal aspect of the thecal sac from L2 to L4 which may represent an abnormal enhancement of a nerve root in the cauda equina. A repeat EMG was indicative of bilateral L3/L4 radiculopathies. At this point, based on clinical, radiographic, and serological testing the patient was diagnosed with HSV2 lumbosacral radiculitis (Elsberg Syndrome) (Fig. 1).

The patient was started on Acyclovir IV 10 mg/kg q8hr for 21 days along with adequate IV fluid hydration, 5 days of high dose methylprednisolone 1 g IV daily, and B12 supplementation 1000 mcg daily. She needed an aggressive pain control regimen which included Tylenol, Flexeril, lidocaine patch, duloxetine, Percocet, and Dilaudid. 10 days after the first LP was performed a second lumbar puncture was done and came back positive for HSV-2 but with no significant improvement of the CSF fluid analysis. 14 days later, a third LP was remarkable for improved WBC count (360 down to 80) and protein level (182 down to 73).

After being an inpatient for three weeks, the patient was discharged to acute inpatient rehabilitation to improve mobility and to maximize safety and functional independence. She remained in rehab for intensive physical and occupational therapy for one month before being discharged home. Her overall duration of symptoms may have also contributed to her symptom improvement. Although she made great progress while being in the acute rehabilitation unit, she was discharged from rehab with a walking cane as a main mode of ambulation.

## Discussion

Elsberg syndrome is a rare cause of infectious lumbosacral radiculitis with a wide variety of clinical presentations and diagnostic criteria. A proposed diagnostic criteria of Elsberg syndrome includes clinical signs and symptoms of caudal equina syndrome, such as urinary/ bowel retention, hesitancy, or incontinence, and MRI or electrophysiologic evidence of cauda equina involvement. Other features that may be suggestive of Elsberg syndrome include preceding genital herpes infection, herpes virus infection in the CSF, clinical or radiographic evidence of myelitis in the conus, acute/subacute onset, and CSF pleocytosis [7]. Our patient met several of the proposed diagnostic criteria as she reported urinary retention, had CSF positive for HSV2, and had MRI evidence of cauda equina involvement as seen on the second MRI taken five days after admission. Additionally, the acute onset of her symptoms and CSF pleocytosis further supported her diagnosis.

Antiviral treatment may affect symptom duration, but there is no evidence it helps with neurologic improvement in herpetic radiculomyelitis. Our patient was started on IV Acyclovir for a total duration of 21 days and received 5 days of high dose corticosteroids. Steroids were initiated to reduce inflammation, and after receiving three days of steroids, the patient began to see improvement in her symptoms. Being cognizant of Elsberg syndrome while treating patients with signs and symptoms of lumbosacral radiculitis is crucial as initiating IV acyclovir for 10–14 days along with corticosteroids is regarded as a treatment of choice due to the possibility of shortening symptoms duration and improving overall morbidity [4].

## Ethical approval

Consent was obtained from patient.

## Funding

Nothing to declare.

## Consent

Written informed consent was obtained from the patient for publication of this case report and accompanying images. A copy of the written consent is available for review by the Editor-in-Chief of this journal on request.

## CRediT authorship contribution statement

**Omar Belfaqeeh:** Writing – original draft. **Alexandria Markley:** Writing – original draft. **Mudita Patel:** Writing – review & editing. **Brian Markoff:** Writing – review & editing, Supervision. **Georgina Osorio:** Writing – review & editing, Supervision.

## Conflict of interest statement

Nothing to declare.
